# Fatal fat embolism in cosmetic surgery: Comparing gluteal fat grafting to other lipofilling procedures

**DOI:** 10.1016/j.jpra.2026.07.006

**Published:** 2026-07-06

**Authors:** Saad Aad, Rawad Hakim, Rateb Saad, Cesar Ghadbane, Hagop Sassounian, Carl Karam, Rawad Rachwan

**Affiliations:** a Faculty of Medicine, University of Balamand, El Koura, Lebanon; b Department of Plastic Surgery, Notre Dame Des Secours University Hospital, Jbeil, Lebanon

**Keywords:** Autologous fat grafting, Aesthetic surgery safety, Lipofilling, Fat embolism, Cosmetic surgery complications

## Abstract

Autologous fat grafting, or lipofilling, is a technique commonly employed in cosmetic/aesthetic procedures for facial, breast, and gluteal augmentation. Although it is considered a safe procedure, fat embolism is a known, albeit rare, catastrophic complication of lipofilling, and gluteal fat grafting is specifically associated with a high mortality rate. This narrative review aims to identify the mechanisms of fatal fat embolism in cosmetic lipofilling procedures and compare the risks of gluteal, breast, and facial fat grafting procedures. Anatomical studies, autopsy reports, and clinical studies suggest that the increased mortality rate associated with gluteal fat grafting is attributable to the interplay of three major contributing factors: the presence of significant intramuscular gluteal veins, the technique of fat grafting, and the amount of fat transferred in gluteal augmentation procedures. In contrast, breast and facial lipofilling procedures are associated with different anatomical environments and injection techniques, which are associated with significantly lower risks of pulmonary fat embolism, with unique risks of arterial fat embolism in facial procedures. The literature has highlighted certain shortcomings in the current body of evidence, including the lack of uniform reporting of complications and the lack of standardized data on procedures performed. It is suggested that enhanced training of surgeons, adherence to strict subcutaneous fat injection, and the use of ultrasound technology in fat grafting procedures, as well as the establishment of mandatory complication registries, are essential strategies for reducing mortality associated with gluteal fat grafting procedures.

## Introduction

Autologous fat grafting known as lipofilling has become one of the most widely performed procedures in cosmetic surgery. Its popularity mainly lies in the dual benefit of contouring a donor site while augmenting a recipient area using the patient's own tissue and avoiding the need for synthetic implants. Lipofilling is currently used in several areas of aesthetic surgery, most commonly in the face, breast, and buttocks. Each of these locations involves different technical approaches, injection volumes, and anatomical considerations.

Despite its versatility, lipofilling is not without serious risk. Among the most feared complications performing this procedure is fat embolism, which occurs when fat lobules or adipose tissue enter the vascular system leading to potentially catastrophic consequences. While fat embolism is known to occur in the setting of trauma, its occurrence in the elective cosmetic and aesthetics surgery context raises a distinct and particular concern, as these procedures are done on otherwise healthy candidates.

This discrepancy in risk between lipofilling sites is not incidental. It could be explained by fundamental differences in vascular anatomy, injection volumes, and differences in techniques between procedures.

The gluteal region contains large-caliber intramuscular venous vasculature in close proximity to typical injection planes, creating a pathway through which fat can rapidly enter the venous circulation and reach the pulmonary circulation. By contrast, facial and breast lipofilling, while not without their own complications, present a markedly different embolic risk profile.

In gluteal fat grafting specifically, fatal pulmonary fat embolism has been identified as the leading cause of procedure-related mortality, with estimated mortality rates of approximately 1 in 3000–6000 cases. This makes gluteal injection’s mortality the highest of any aesthetic procedure.^1^

This review aims to examine the pathophysiology of fatal fat embolism in the context of cosmetic lipofilling, with particular focus on gluteal fat grafting, through a comparative analysis of gluteal, breast, and facial fat transfer. Our goal is to highlight why the buttocks represent such a uniquely high-risk site, and what anatomical and technical factors drive this risk.

## Overview of lipofilling procedures

Autologous fat grafting (lipofilling) follows a reproducible sequence of harvest, processing, and placement, yet each step is adapted to the recipient site and must balance volume goals with vascular safety.^2^^,^^3^ Tumescent infiltration precedes the harvesting stage and is done using low-trauma aspiration; free-oil contamination and adipocyte rupture are increased in excessive negative pressure, prolonged exposure to air, and small-bore instrumentation.^4^ Next comes processing which reduces fluid and blood and free lipid via either decantation, washing or centrifugation. This step ensures a graft with consistent particle size and minimal inflammatory debris. Graft may be enriched using protocols such as cell-assisted lipotransfer using stromal vascular fraction, but techniques are heterogeneous and regulated variably across jurisdictions,^5^^,^^6^ with closed-system transfer when feasible.

Placement generally is the retrograde low-pressure injection of small aliquots using blunt cannulas. Injections are distributed through multiple tunnels (“microdroplet/threading”) to reduce bolus necrosis and increase surface-area contact permitting revascularization.^2^ In the face, injections are of smaller volumes and frequently compartment-based; variable retention was shown in pooled analyses supporting counseling and staged correction.^7^^,^^8^ Separately, for intradermal use and skin quality rather than bolus volume replacement, emulsified “nanofat” preparations are used.^9^ Catastrophic ocular or cerebral embolic events after facial fat injection have been described although these events are rare, emphasizing anatomic risk zones and avoidance of intravascular placement.^10^^,^^11^

In the breast, lipofilling is used for cosmetic enhancement and as an addition in reconstruction or treatment of radiotherapy injury. This is commonly done in staged sessions to avoid excessive recipient-site pressure and account for partial resorption.^12^^,^^13^ Oncologic safety in appropriately selected patients is described in meta-analyses, although common local issues include oil cysts, fat necrosis ad imaging changes necessitating biopsy or interval mammography.^12^^,^^14^ Gluteal fat grafting differs mostly in the volume transferred and the proximity to large deep veins; mean injections around several hundred milliliters per side, frequently combining subcutaneous and intramuscular planes, has been reported by systematic review data.^15^ Ultrasound-guided and advisory-based approaches emphasize subcutaneous-only injection and documentation of cannula plane, with prospective ultrasound analysis demonstrating durable subcutaneous thickness gains at 12 months.^16^, ^17^, ^18^

## Pathophysiology of fatal fat embolism

Gluteal fat grafting is the most strongly associated with fatal fat embolism, especially when the cannula violates the gluteus maximus, injuring large veins or venous plexuses and allowing lipoaspirate to enter as macroscopic fat into the venous system.^1^^,^^19^ Abundant fat within injected muscle and concurrent pulmonary microembolism are reported in autopsy-based series, with macroscopic emboli in the most fulminant cases.^20^^,^^21^ No area is completely safe from vascular injury in injections and this is supported by imaging-based anatomy demonstrating that superior and inferior gluteal veins can lie immediately deep to gluteus maximus.^22^ The risk of penetrating vessels is further increased with smaller cannulas, downward angulation and high injection pressures.^1^^,^^16^ Importantly, record-review data suggest that the best predictor for lethality is the presence of macroscopic fat in circulation and not the total volume injected.^20^

Once fat reaches the venous circulation it rapidly traverses the right heart and impacts the pulmonary arterial tree, producing sudden ventilation–perfusion mismatch and an abrupt rise in pulmonary vascular resistance, which can precipitate acute right ventricular failure, hypoxemia, and cardiovascular collapse.^20^^,^^21^ Fatal events usually occur intraoperatively or within minutes after surgery with hypoxemia, hypotension and bradycardia.^19^^,^^20^ Fat embolism is a leading mechanism of death as reported in forensic series of elective gluteal procedures and as proved by autopsy findings revealing gross fat withing pulmonary vasculature and widespread microemboli.^21^ Mechanical obstruction appears dominant in these catastrophic cases, explaining the ineffectiveness of rescue once instability begins. Accordingly, prevention strategies should be used by avoiding deep planes where large veins are prevalent and injecting in the subcutaneous areas.^16^^,^^17^

Fat emboli are common after long-bone trauma but the clinical Fat Embolism Syndrome (FES) is less frequent and often occurs after 24 to 72 h with the triad of respiratory insufficiency, neurologic disturbance, and petechial rash.^23^ Trauma FES is commonly viewed as mix of mechanical and biochemical damage: marrow-fat microdroplets embolize, then undergo lipolysis to free fatty which contribute to endothelial dysfunction, inflammation, and ARDS-like physiology.^24^^,^^25^ With appropriate hemodynamic and respiratory care, patients survive and management is mainly supportive.^25^ On the other hand, fatal embolism is immediate and intraoperative during gluteal lipoinjection without a prodromal window, consistent with massive macroscopic embolization rather than delayed inflammatory amplification.^19^^,^^20^ This distinction has important clinical implications: preventive measures in lipofilling rely less on early identification and more on avoiding damage to veins and restricting injections to verified safer layers.^1^^,^^16^

### Comparative synthesis tables and diagram

To better explain the differences in embolic risk between lipofilling procedures and to clarify the mechanism of fatal fat embolism in cosmetic surgery, two comparative tables are presented below (Table 1, Table 2).

**Table 1:** Procedural contrasts relevant to systemic embolic risk (facial vs breast vs gluteal lipofilling).

Characteristic	Facial Lipofilling	Breast Lipofilling	Gluteal Lipofilling
Typical volume per session	Small; multi-compartment aliquots (generally <100 mL total)	Moderate; typically 100–300 mL per breast, often performed in staged sessions	Large; approximately 400–700 mL per side, sometimes exceeding 1000 mL
Injection plane	Compartment-based; deep fat and supraperiosteal layers	Multi-plane subcutaneous injections with layered microinjection technique	Historically mixed planes; current guidelines recommend subcutaneous-only injection
Primary vascular danger	Arterial — retrograde embolism to retinal or cerebral vessels	Minimal systemic vascular risk; complications are usually local	Venous — direct entry of fat into large intramuscular gluteal venous channels
Fatal pulmonary embolism reported	Rare	Extremely rare with only isolated case reports	Yes — recognized as the leading cause of procedure-related mortality
Key safety concern	Anatomical danger zones close to ophthalmic arterial connections	Oncologic safety, radiologic changes on imaging, and fat necrosis	Control of injection plane, cannula angulation, injected volume, and use of ultrasound guidance

Sources: facial retention and complication syntheses,^7,10,11^ breast evidence syntheses and radiotherapy applications,^5,12–14^ gluteal technique patterns and ultrasound-supported plane control.^15–17^

**Table 2:** Mechanistic distinction: catastrophic pulmonary fat embolism during gluteal lipoinjection vs trauma-associated FES.

Characteristic	Fatal Embolism in Gluteal Fat Grafting	Trauma Fat Embolism Syndrome (FES)
Typical Onset	During the procedure or within minutes after fat injection	Delayed onset, typically 24 to 72 h after traumatic injury
Type of emboli	Macroscopic fat boluses entering large venous channels	Microscopic marrow fat droplets released from fracture sites
Main mechanism	Mechanical obstruction of pulmonary arteries leading to acute right heart failure	Combination of mechanical microembolism and inflammatory response from free fatty acids
Clinical presentation	Sudden cardiovascular collapse with no preceding warning signs	Progressive triad consisting of respiratory distress, neurological symptoms, and petechial rash
Outcome without intervention	Rapidly fatal in many cases; successful resuscitation is uncommon once collapse occurs	Frequently survivable with adequate supportive care
Primary clinical strategy	Prevention through safe surgical technique and avoidance of venous injury	Early recognition and supportive management including ventilation and hemodynamic stabilization

Sources: gluteal mortality and autopsy/record series,^1,19,20^ trauma-FES framing.^1,24,25^

Together, these tables highlight two key ideas: first, that the gluteal region represents a unique anatomical setting that increases the risk of intravascular fat injection, and second, that once a massive cosmetic fat embolism occurs, treatment options are extremely limited, making prevention the most important strategy.

## Site by site comparison

### Gluteal fat grafting

Mortality estimates from the ASERF Task Force place gluteal fat grafting at approximately 1 in 3000–6000 procedures making it the highest reported mortality of any aesthetic procedure with pulmonary fat embolism being the principal cause.^1^ This is reflected in the significantly higher incidence of fatal and non-fatal pulmonary embolic events reported after gluteal fat grafting when compared with other procedures involving fat transfer or manipulation of adipose tissue.^1^

What distinguishes the gluteal region, as demonstrated by cadaveric and imaging studies, is the presence of large-caliber venous channels within the intramuscular planes of the gluteus maximus, some measuring several millimeters in diameter, as well as thin-walled venous channels embedded within muscle fibers.^19^ These vessels may be particularly vulnerable during fat grafting procedures when injections are performed into deep tissue planes using long cannulas. Intramuscular injections performed with steep downward angulation of the cannula may bring the cannula tip in close proximity to these veins. Because the course and depth of these vessels can be highly variable, they may be difficult to reliably avoid during deep injections. Disruption of the vessel wall during injection may therefore allow adipose tissue to enter the venous circulation and subsequently drain into the internal iliac venous system, through which embolic material can rapidly reach the pulmonary circulation.^1^

Another factor contributing to the increased risk of embolization is the relatively large volume of transferred tissue used in gluteal augmentation. Injection volumes in gluteal fat grafting commonly range from 300 to 700 mL per buttock, although larger transfers exceeding 1000 mL have been reported in some series.^26^

For these reasons, the ASERF Task Force issued several recommendations aimed at reducing the risk of fat embolism during gluteal fat grafting. These include avoiding injections into the deep muscle, using blunt cannulas with a diameter of at least 4.1 mm, and avoiding downward angulation of the cannula during injection.^1^

### Breast lipofilling

The challenges posed by the unique anatomy of the gluteal region can be further highlighted by comparing gluteal fat transfer with breast lipofilling. The latter carries a significantly lower risk of systemic embolization. These structures lack the large intramuscular venous channels found in the gluteal region that are critical in the pathophysiology of fat embolism.

Another point of comparison is the volume of adipose tissue transferred during breast lipofilling. Injection volumes used in breast fat grafting are generally smaller than those used in gluteal augmentation. Typical transfers range from approximately 100–300 mL per breast, and fat is usually injected in multiple small aliquots using a layered microinjection technique designed to maximize graft survival while minimizing tissue trauma.^27^ As a result, the likelihood of fat entering the systemic circulation is significantly reduced.

Consequently, pulmonary fat embolism following breast lipofilling appears to be rare. Systematic reviews indicate that the vast majority of embolic events occur following gluteal procedures rather than breast fat transfers.^28^

Nevertheless, isolated cases of pulmonary fat embolism have been reported,^10^ describing a patient who developed acute respiratory compromise shortly after receiving approximately 270 mL of fat per breast during cosmetic fat grafting, demonstrating that systemic embolization remains a rare but possible complication.

### Facial fat grafting

Unlike gluteal and breast fat transfers. Facial fat grafting presents a fundamentally different risk. With most emboli arising from arterial circulation and not venous ones. This is due to the underlying extensive vasculature with numerous branches of the external carotid artery anastomosing with ophthalmic circulation. As a result, inadvertent injections of adipose tissue into the vessels risk sending emboli into the cerebral or ophthalmic circulation.

These events may result in severe complications including retinal artery occlusion, vision loss, or cerebral infarction. In contrast to gluteal fat grafting, where embolic material typically enters the venous circulation and subsequently reaches the pulmonary vasculature, embolic events following facial fat grafting are primarily arterial in origin.^29^^,^^30^

Likely due to the small volume of fat transferred usually totaling a few millimeters of fat delivered in small aliquots within superficial tissue planes.^2^

## Risk factors

Fatal fat embolism in gluteal fat grafting is primarily related to disruption of large intramuscular veins that belong to the inferior and superior gluteal venous systems during deep fat injection. When these vessels are penetrated, adipose tissue may enter the venous circulation directly. Once fat reaches the venous system, it can rapidly travel to the pulmonary circulation, producing massive pulmonary embolization and potentially resulting in acute right heart failure.^31^

According to a recently performed systematic review, all reported cases of fatal fat embolism were related to intramuscular injections into the gluteal area, while subcutaneous injection procedures were not associated with any reported fatalities during fat grafting procedures.^32^

Injection technique and cannula position are also of great importance in the process. For example, the downward angulation of the cannula will make it more likely to penetrate the gluteal fascia and enter the muscle layer. Fat grafting is performed without visual control of the cannula tip, and as a result, it is possible for the cannula to penetrate too deeply, especially when using high injection pressure.^31^ High injection pressure can cause fat to enter injured vessels or disrupted venous sinusoids as well. Another factor is the large volumes of fat transferred in fat grafting procedures, which is a characteristic of gluteal augmentation procedures. For example, compared with facial and breast lipofilling procedures, gluteal lipofilling procedures require the transfer of hundreds of milliliters of fat for each gluteal area. Large volumes of fat are associated with increased duration of injection and pressure on tissues, and as a result, they are more likely to cause vascular injury and fat embolization.^33^ Other contributing factors are the use of sharp cannulas, which are more likely to cause vascular injury, and the use of high-force lipoinjection techniques. According to a review of fat embolisms associated with liposuction and fat grafting procedures, almost 70% of fat embolisms resulted from using high-force injection and large-volume fat transfer.^32^ Patient-related factors are also of great importance in the process, and they include high body mass index, concurrent procedures, and vascular abnormalities; however, they are still secondary to procedural variables in determining fatal outcomes.^34^

Overall, the convergence of intramuscular injection, high-volume grafting, and blind cannula placement creates a procedural environment in which venous injury and systemic fat embolization are more likely. These risk factors largely explain why fatal pulmonary embolism is disproportionately associated with gluteal fat grafting compared with other lipofilling procedures.

## Prevention

Prevention of fatal fat embolism in autologous fat grafting is ensured by employing surgical techniques that reduce the likelihood of fat particles entering the venous system. In the past few years, professional and regulatory bodies have issued guidelines on fat grafting safety, based on clinical and anatomical evidence that has accumulated in this regard.^16^ The most important method in preventing fat embolism is limiting fat injection to the subcutaneous plane alone. Recent guidelines on fat grafting safety emphasize the need to inject fat only in areas that are superficial to the gluteal fascia, in order to avoid the intramuscular venous system, which is known to cause most fat embolisms.

Blunt, large-diameter cannulas are also strongly supported, as they are less likely to injure vessel walls and allow more controlled fat distribution, reducing the risk of intravascular entry.^31^ A recent advancement in the field of fat grafting has been the use of intraoperative ultrasound guidance. Systemic studies and meta-analytic reviews published between 2023 and 2025 reported extremely low rates of complications during fat grafting when intraoperative ultrasound was employed. No fat embolisms and fat grafting-related mortality were reported in the studies. Due to the results of the studies mentioned above, the use of intraoperative ultrasound has now been made mandatory during fat grafting procedures in the gluteal area.^26^

Ultimately, prevention of fatal fat embolism depends on adherence to evidence-based procedural protocols. Current literature suggests that maintaining a subcutaneous injection plane, using blunt cannulas, employing ultrasound guidance, and improving surgeon training represent the most effective strategies for reducing mortality associated with gluteal fat grafting.

## Discussion

The evidence synthesized in this review confirms that fatal fat embolism risk is concentrated in gluteal fat grafting, driven by the convergence of three factors: anatomy, technique, and volume.

Anatomical studies have clearly identified that the gluteal region is uniquely prone to the problem of venous injury and the resultant macroscopic fat embolism due to the significant caliber of the veins that traverse the gluteus maximus muscle and the deep tissues that lie below it, including the superior and inferior gluteal venous systems, which can be as large as several millimeters in diameter and can be embedded in the muscle tissue itself, making it particularly prone to the problem of cannula penetration.^19^^,^^22^ The autopsy results of individuals who have died as a result of the procedure have clearly identified the presence of fat within the gluteus maximus muscle and the macroscopic fat embolus in the pulmonary circulation, which is consistent with the theory that the injury to the venous system has allowed the macroscopic fat to enter the systemic circulation.^20^^,^^21^ Once the macroscopic fat has entered the systemic circulation, it is rapidly carried through the right heart and into the pulmonary circulation, where it can cause mechanical obstruction and cause acute increases in pulmonary vascular resistance, which can cause acute failure of the right heart and the resultant cardiovascular collapse.

Furthermore, technical factors are also known to contribute to this anatomical susceptibility. Indeed, fat grafting in the gluteal area is frequently carried out using long cannulas inserted through several tunnels and at steep angles in the direction of the floor. In such cases, the tip of the cannula passes through the fascia and into the muscular tissue. It has the potential to come into direct contact with the venous plexa present in the muscular tissue. High injection pressures and large volumes injected as a bolus are known to force fat into damaged vessels and disrupted venous sinusoids.^1^ Moreover, the absence of direct visualization makes it very difficult to control the depth of the cannula. This is in stark contrast to other fat injection procedures in which the fat is injected in the superficial planes.

Volume injected is the third factor in the fat injection procedures in the gluteal area that has resulted in the mortality rates in this area. Indeed, fat injection in the gluteal area involves the injection of several hundred milliliters of fat per side. In fact, the volume injected in this area has been reported to be as high as 400–700 mL.^15^ This results in the increase in the time required for the injection and the mechanical pressure exerted during the fat injection. Autopsy results have shown that the presence of fat in the lung tissue is the strongest predictor of fat injection fatality.^20^

In contrast, breast and facial lipofilling present fundamentally different risk profiles rather than being simply lesser versions of the gluteal risk. Breast tissue lacks large intramuscular venous channels; fat is injected in smaller volumes across superficial planes, making pulmonary embolism rare. Complications in breast lipofilling are predominantly local: fat necrosis, oil cysts, and imaging changes.

Facial fat grafting carries a distinct vascular risk profile driven by the arterial rather than the venous system. The anastomoses between the external carotid and ophthalmic vessels create a pathway for retrograde arterial embolism leading to different consequences including vision loss and cerebral infarction rather than pulmonary complications.

Despite the increased recognition of this complication, there is still a significant gap in the literature. Firstly, it is difficult to obtain reliable data on the incidence of the complication because the reporting is not uniform and is often voluntary. The estimates of mortality rates, as reported by the ASERF Task Force, are based on voluntary reporting and not on any registry data, which can be prone to underreporting and reporting bias.^1^ Secondly, there is still a lack of sufficient scientific evidence as many studies have reported individual case series and autopsy results, which although provide valuable insights into the mechanisms, do not help in quantification and determining the risk involved in the procedure across different settings. Thirdly, the technical aspects involved in the procedure, such as the design of the cannula, the pressure required for injection, the experience of the surgeon, and the use of intraoperative imaging, are not standardized across studies and settings, making it difficult to determine the contribution of each factor towards the causation of the fatal outcome.

To bridge the gap, there is a need to make significant changes to the way the procedure is carried out and the way the research is conducted. Firstly, there is a need to standardize the procedure and improve the training methods and skills of the surgeons. The recommendations that have been proposed and emphasized to improve the safety of the procedure include the injection of fat into the subcutaneous tissue alone, the use of blunt-tipped cannulas with larger diameters, the avoidance of angulating the cannula towards the floor, and the use of ultrasound to guide the procedure and confirm the plane of injection.^16^ Secondly, the regulatory bodies can be instrumental in improving the safety of the procedure through the issuance of guidelines and safety alerts on the procedure and the mandatory use of ultrasound during the procedure in the country, which has now become mandatory to ensure that the injection is carried out in the subcutaneous tissue alone and not in the gluteal region, as the former has fewer venous channels compared to the latter.

Lastly, better reporting systems are crucial in the accurate determination of the epidemiology of fat embolisms in aesthetic surgery. Mandatory complication registries and reporting systems can be very effective in ensuring better risk assessment and in determining the procedures that are most likely to cause complications. Without this information, the mortality and complication rate can only be estimated, and this can be very limiting in the implementation of evidence-based safety guidelines.

## Conclusion

The most severe complication related to autologous fat grafting is the fatal fat embolism, which is seen predominantly with gluteal fat grafting compared to other types of lipofilling. From the evidence reviewed in the study, it is seen that the increased risk of this type of fat grafting is due to the coincidence of three main factors related to the unique venous anatomy, the technical aspects of the procedure, and the volume of grafting required in the case of gluteal augmentation compared to other types of lipofilling. The risk factors related to breast and facial lipofilling show unique profiles compared to the risks seen with gluteal fat grafting due to the unique anatomy and the volume required in the procedure. Therefore, the complications seen with these types of fat grafting are different compared to the risks seen with gluteal augmentation. Though the risks and complications related to the procedure have gained significant attention, there is still much to be addressed in the literature with regard to adverse effects and the technical variables related to the procedure that could be improved to reduce the risk of fatal fat embolism in the procedure.

## Funding

This research project received no funding.

## Artificial intelligence use statement

During the preparation of this work the authors used **ChatGPT (OpenAI)** in order to assist with **language editing and structural refinement of the text**. After using this tool, the authors reviewed and edited the content as needed and take full responsibility for the content of the publication. No AI tools were used for data generation, analysis, or interpretation.

## Ethical approval

Not required.

## Declaration of competing interest

Authors have nothing to declare.
